# Editorial: Innovative Therapeutic and Immunomodulatory Strategies for Protozoan Infections

**DOI:** 10.3389/fcimb.2019.00293

**Published:** 2019-08-09

**Authors:** Jorge Enrique Gómez Marín, Kamal El Bissati

**Affiliations:** ^1^Grupo GEPAMOL, Centro Investigaciones Biomédicas, Facultad de Ciencias de la Salud, Universidad del Quindío, Armenia, Colombia; ^2^Institute for Molecular Engineering, University of Chicago Medical Center, Chicago, IL, United States

**Keywords:** Protozoa (source: MeSH), *Toxoplasma*, *Leishmania*, *Trypanosoma*, *Plasmodium*, immunomodulation, synthetic vaccines, systems biology parasitic sensitivity tests

In this special issue of Frontiers in Cellular and Infection Microbiology, we assembled a collection of 9 original research articles and 3 reviews within the theme “Innovative Therapeutic and Immunomodulatory Strategies for Protozoan Infections.” The intended goal of this Research Topic was to present an updated view of current innovations for intervention strategies against human protozoan infections. No completely efficacious vaccines for human protozoan infections exist. For example, the current vaccines licensed for malaria, or those currently being investigated in clinical trials, are not completely effective and their utility are controversial (Genton, 2008; Thera and Plowe, 2012). This same limitation exists with respect to availability of drugs to treat infected individuals. Often wholly effective medications are lacking, and effective medications are suboptimal. New challenge is posed by artemisinin-based combination therapy (ACT)-resistant strains of *P. falciparum*, making development of new drugs to save malaria patients ever more urgent. In ocular toxoplasmosis, complete eradication of the parasite from the host is not possible, exposing patients to persistent threat of disease reactivation (de la Torre et al., 2009). Pyrimethamine and sulfadiazine, with folinic acid, are the mainstays of treatment for toxoplasmosis, but do not treat the latent bradyzoite life-stage. In the case of South American trypanosomiasis (Chagas disease) only two drugs (benznidazole and nifurtimox) compose the armamentarium of physicians treating patients with this disease (Gomez-Marin, 2010). In light of these challenges, new research strategies have appeared that enhance the possibility of treatment and vaccine development including reverse vaccinology (Cardona et al., 2015), the bioinformatic search of second-use drug candidates (Osorio and García, 2019), and *ex vivo* analysis of immune response (Torres-Morales et al., 2014). This Research Topic evaluates these new possibilities and brings together authorities in this field and experiences that will feed the next wave of drugs and vaccines for this group of infections that have significant human health consequences. In toxoplasmosis, Acosta Davila et al. reviewed and summarize the utility of peripheral blood mononuclear cells (PBMCs) as a model to investigate the immunologic component of host-parasite interaction. It also delineates technical limitations associated with handling and subsequent processing of PBMCs. The authors highlight the importance of variables (e.g., time of collection of PBMC), significant implications for data interpretation and conclusions related to host-parasite interaction, and discuss some controversies related to this model and suggest possible improvements to related protocols. This *ex vivo* model of infection is critical to obtain accurate information about human response to infection, given notable differences in immune response in mice (Gómez-Marin, 2000). Critical to the development of new therapies against *T. gondii*, Rocha-Rao et al. reported the use of a thiazolodinone scaffold and its derivatives, all of which contain an imidazole ring, and how these compounds may lead, in the future, to improved therapies targeted against the parasite. The authors discuss in their manuscript different scaffolds that they studied and how improvements might be made, especially in terms of *in silico* design. Evaluation of kinase inhibitors represents a novel strategy that may overcome difficulties of cross-inhibition of host kinases. The paper of the Argentinian group, led by López et al., demonstrated interesting compounds that merit further exploration *in vivo*. In this work, proteins involved in DNA replication or repair are proposed as potential therapeutic targets of human pathogens. The authors provide evidence demonstrating the effect of phosphatidylinositol 3-kinase (PI3K) inhibitors (KU-55399), showing that they block *Toxoplasma* tachyzoite replication. They found an abolition of the phosphorylation of H2A.X, proposing that ATM kinase can be a potential drug target for abrogation of *T. gondii* growth. In the same vein, an international interdisciplinary collaborative team including the University of Chicago, the Center for Structural Genomics of Infectious Diseases at Northwestern University, and collaborators across the U.K., provides crystallographic structures of 5 *Toxoplasma* small enzymes function in key metabolic pathways likely to be vital for parasite survival for the international research community to use (Lykins et al.). Moreover, the group demonstrates that target-specific modulation via morpholino inhibited parasite replication *in vitro*. The authors hope that obtaining crystal structures of these enzymes will facilitate discovery of drugs against *Toxoplasma*. Looking for new compounds that block *Toxoplasma* at the bradyzoite stage (responsible for disease reactivation across time) remains a challenging task for scientists. Vaccine development, therefore, represents a reasonable approach to would prevent infection from becoming established. Considerable progress has been made toward identification of vaccine prototypes that are moving closer to clinical studies. This includes recombinant protein that elicits CD8^+^ and CD4^+^ T cells response in HLA transgenic mice (El Bissati et al., 2016); Self Assembling Protein Nanoparticles (SAPN) (El Bissati et al., 2014, 2017). RNA replicons and liposomes, amongst other methods, are other vaccine platforms reported in the literature to improve mouse survival against *Toxoplasma* challenges. One of the most significant challenges in vaccine development is to trigger adequate antigen presentation, requiring not only sufficient quantity of antigen, but also a stable preparation, facilitating a durable immune response. Guo et al. reported use of chitosan microspheres as a system that offers these properties and confers protection to mice against acute and chronic toxoplasmosis. The authors employed bioinformatics to screen predominant peptides that bind to T and B cells epitopes. The ones that elicited IgG were selected to compose a multigenic peptide, emulsified with chitosan and microspheres, allowing assessment for immunogenicity. Similar efforts on antigenic selection was used by Patarroyo's group from Bogota (López et al.). In this work, B- and T-cell epitopes of the *P. vivax* rhoptry neck protein 2 (PvRON2) were predicted *in silico* and then, the binding of predicted epitopes to HLA-DR molecules was experimentally assessed, with four peptides selected to test immunogenicity (i.e., T cell proliferation, cytokine production, and IgG production) in cells from *Plasmodium vivax*-exposed individuals from two endemic areas in Colombia. Authors concluded that two T-cell epitopes and one B-cell epitope are promising vaccine candidates. This work illustrates the difficulties to drawing conclusions from immunological assays with human samples. In addition, Alti et al. reviewed current knowledge about the use of leptin as adjuvant. The role of leptin in immune regulation is an interesting topic that should be considered given its implications on the efficacy of vaccines in obese individuals, suggesting this factor should be included in evaluations of vaccine efficacy.

Strategies of treatment against other protozoans were also reported in this Research Topic. In malaria, given the discovery of strains of *Plasmodium* with intrinsic resistance to the very efficacious artemisinin (Aponte et al., 2017), new drugs are urgently needed. El Bissati et al. explored the therapeutic potential of targeting the polyamine pathway in *Plasmodium* and, in particular, spermidine synthase as a target of a new family of inhibitors. The polyamine pathway appears to be a valid target for further exploration and small molecule development, given its essential role in parasite survival, is likely to bear therapeutic fruit. Testing analogs effective against Microsporidia formed the basis of the authors' initial approach toward *P. falciparum* studies. Tetraamines and oligoamines have previously documented efficacy in experimental murine models of microsporidiosis. Surprisingly, these compounds showed no or minimal antimalarial activity. The authors found that addition of cyclohexylmethyl side groups to the tetramine backbone conferred potent inhibitory activity in both chloroquine-resistant (Dd2) and chloroquine-sensitive (3D7) strains at the low nanomolar range. Furthermore, these polyamine analogs decreased levels of spermine in infected cells, suggesting a mechanism involving inhibition of spermidine synthase.

Urban transmission of trypanosomiasis and new foci for Chagas' transmission in South America have increased the interest in development of optimized treatment approaches (Rueda et al., 2014; Bello Corassa et al., 2016). The review by Osorio-Méndez and Cevallos offers perspective regarding how this might be achieved. The authors use current genetic and bioinformatics tools for identifying new molecular targets for chemotherapeutic treatment of *T. cruzi*, the causative agent of Chagas disease, and follow up with in-depth descriptions of possible targets enzymes in three metabolic pathways: synthesis of surface glycoconjugates, ergosterol biosynthesis, and neutralization of reactive oxygen species. Alberca et al. chose to focus their work on an alternative pathway, instead using a virtual ligand and structure-based screening to find polyamine analogs that could bind to the *T. cruzi* polyamine transporter putrescine permease TcPAT12. From the large libraries submitted for docking analysis, the authors found several hits with activities against *T. cruzi* and conclude that virtual screening is effective in finding inhibitors of putrescine uptake in *T. Cruzi*. In another study Gunjan et al., have studied the effect of artemisinin derivatives (α/β artemether, artesunate, and a synthetic 1, 2, 4 trioxane) on apoptotic pathway of malaria parasite and its survival. Finally, the work of the Mexican group on *Giardia*, Argüello-Garcia et al. and garlics derivatives, is an *excel sum* example about how nature continues offering new possibilities to find anti parasite compounds.

In conclusion, this Research Topic is a good sample of how novel chemoinformatics and bioinformatics strategies are revolutionizing the discovery and identification of new compounds. The new tools such as CRISP arrayed libraries of mutated genes have added recently new possibilities and we have seen recently an explosion of new anti-protozoa compounds. However, there is still the barrier to test such a large number of compounds, the same is occurring with the vaccines candidates and it is necessary to develop efficacious selective screening methods for new drugs and vaccines. At this respect, the organoids and tissue explant-based models of infection can offer a new strategy to be developed for the evaluation of anti-protozoan drug candidates (Broutier et al., 2017). This could represent a pathway of hope to obtain better drugs and vaccines for these important human diseases.

## Author Contributions

JG and KE drafted and edited the editorial.

### Conflict of Interest Statement

The authors declare that the research was conducted in the absence of any commercial or financial relationships that could be construed as a potential conflict of interest.
